# Moral Distress and the Cost of Caring Amongst Medical Oncologists in Singapore

**DOI:** 10.1177/10499091251330607

**Published:** 2025-03-27

**Authors:** Varsha Rajalingam, Yutian Yu, Yun Ting Ong, Annushkha Sinnathamby, Nila Ravindran, Nagavalli Somasundaram, Simon Yew Kuang Ong, Lalit Kumar Radha Krishna

**Affiliations:** 1 Yong Loo Lin School of Medicine, National University of Singapore, Singapore; 2 Division of Supportive and Palliative Care, 68751 National Cancer Centre Singapore, Singapore; 3 Division of Cancer Education, 68751 National Cancer Centre Singapore, Singapore; 4 Khoo Teck Puat National University Children’s Medical Institute, National University Health System, Singapore; 5 Division of Supportive and Palliative Care, 203378 National University Cancer Institute Singapore, Singapore; 6 Division of Medical Oncology, 68751 National Cancer Centre Singapore, Singapore; 7 Duke-NUS Medical School, National University of Singapore, Singapore; 8 Centre for Biomedical Ethics, National University of Singapore, Singapore; 9 Palliative Care Institute Liverpool, Academic Palliative & End of Life Care Centre, Cancer Research Centre, 4591 University of Liverpool, Liverpool, UK; 10 PalC, The Palliative Care Centre for Excellence in Research and Education, Singapore; 11 Health Data Science, 4591 University of Liverpool, Liverpool, UK

**Keywords:** moral distress, compassion fatigue, burnout, vicarious trauma, oncology, end of life care, secondary traumatic stress

## Abstract

**Background:**

Moral distress (MD), the notion of not being permitted to act in a morally and ethically correct manner, is evident amongst oncologists caring for terminally ill patients. Oncologists often contend with complex decisions, such as withholding treatment and managing family distress. Sociocultural and individual considerations also influence an oncologist’s perception of MD, which can vary in severity due to changing ethical, practical, clinical, moral and professional considerations and shifting contextual circumstances. Their impact compromises an oncologist’s wellbeing, patient outcomes and care of their family. Recent data suggests long-term consequences to MD and alludes to a broader cost of caring that encompasses compassion fatigue, vicarious trauma, secondary traumatic stress and burnout.

**Methods:**

This study aims to determine how oncologists in Singapore experience MD and the costs of caring through secondary analysis of interviews with 12 oncologists.

**Results:**

Analysis of the interview transcripts revealed the following domains: 1) sources of MD; 2) sources of the costs of caring; and 3) protective factors.

**Conclusion:**

This secondary analysis of Singaporean oncologists suggests that MD is not frequently an isolated experience; rather, it leads to growing distress amongst oncologists—contributing to a wider cost of caring. This then impacts oncologists’ decisioning, actions, practice and career trajectories. Longitudinal structured training, establishing personalised support for all oncologists and creating a safe working environment supported by the host organisation are thus critical to ensure sustainable practice.

## Introduction

Experiences during the COVID-19 pandemic have had significant ramifications upon the careers, mental health and general wellbeing of physicians.^1^ These reports have refocused attention to the impact of moral distress (MD) on physicians.^2^ However, effective study of this phenomenon is compromised by the lack of a clear understanding of MD.

MD has been variously attributed to *“a serious threat to an individual’s moral integrity, inability to act on core values and obligations”*^3^; *“distress resulting from one’s actions not achieving the desired outcome”*^4^; and deciding on a course of action without effectively balancing the issues or a clear determination of what is ethically right.^5,6^ In truth, concepts of MD have been shown to be shaped by the physician’s prevailing values, beliefs and principles (henceforth belief systems), as well as the ethical, practical, clinical, moral and professional considerations in the wider sociocultural setting.^3,5-10^ It is also affected by resource availability, manpower shortages, high workloads and poor continuity of care.^3,6,7,10,11^

Recent studies have revealed that physicians not only experience MD, but also a wider cost of caring that encompasses compassion fatigue, vicarious trauma and secondary traumatic stress.^12-15^ If left unattended, this may predispose to burnout and limit physicians’ practice and ability to find meaning and satisfaction in their roles. This is especially felt by medical oncologists (henceforth oncologists) due to their involvement in end-of-life care; recruitment of terminally-ill patients in clinical trials; use of therapies based on extrapolated data and questionable evidence; and acquiescence to unwise patient choices and family demands.^16-21^

Contributing to the above phenomenon in Singapore are ‘Asian values’ transmitted by local governmental policies rooted in regnant family-centric thinking.^22,23^ These evolved Confucian-based concepts have been absorbed by a multi-cultural, multi-ethnic and multi-religious population^24^ that considers the interests of the family unit as part of, or more important than, the individual. This is partly due to the belief that the fate of the individual impacts the family as a whole,^25,26^ leading to familial participation in any decision-making processes.^27-29^ However, taken to the extreme, familial determination may usurp the patient’s autonomy and right to self-determination.^27-30^ It also reduces personal privacy as family members become privy to any and all aspects of care determinations. In some cases, this dynamic dispenses with the involvement of the patient as a whole.^27,28,31,32^ Moreover, the patient’s best interests might be trumped in favour of those determined by the family,^27,28,33,34^ leading to practices such as collusion. These scenarios can significantly impact care approaches and end-of-life practices locally and regionally, such as in Malaysia, Taiwan, Hong Kong, China, Korea and Japan.^28,30,35,36^

One such example illustrating the complex interplay of cultural factors and oncological treatment principles is the act of providing treatments, such as total parenteral nutrition (TPN), to conform with familial beliefs and societal norms on feeding at the end-of-life. This is despite regnant clinical evidence^37^ suggesting the limited impact of such interventions on the prognosis of patients at the end-of-life. Rather, such interventions may even diminish the quality of life, prolong the dying process and impede the patient’s wishes to pass at home. Contextually, however, feeding at the end-of-life is a key sociocultural belief in Singapore. These beliefs purport feeding as a sign of ‘love’, non-abandonment and enduring filial piety.^18,38,39^ Continuing to feed loved ones via artificial means, including TPN, intravenous hydration or nasogastric tubes, presents a sign of ‘maintaining hope’—another key facet in local interpretations of filial piety underpinning Asian values.

Individual and contextual factors thus influence MD and its links with the wider costs of caring, burnout,^10^ job dissatisfaction, increased intention to quit^5,10,11,40,41^ and compromises to physicians’ physical and mental health.^42^ Considering the downstream effects on patient care, outcomes, experiences and safety, there is a pressing need to better appreciate MD, the costs of caring and the precise mechanisms by which individual, contextual and sociocultural factors exert their influence.

## Methods

To answer the primary research question, *“What is known about moral distress and the costs of caring on medical oncologists in Singapore?”*, and secondary questions including, *“What are the factors contributing to moral distress and the costs of caring on medical oncologists in Singapore?”; “What is the impact of moral distress and the costs of caring on medical oncologists in Singapore?” and “How do medical oncologists in Singapore cope with moral distress and the costs of caring?”*, we carried out a secondary analysis of previously conducted semi-structured interviews with oncologists with MD at a tertiary cancer centre in Singapore.^43^

### Ethics Approval and Consent to Participate

Ethics approval (2021/2176) was obtained from the SingHealth Combined Institutional Review Board. Informed written and verbal consent was obtained from all participants.

### Semi-Structured Interviews

A systematic approach was adopted by the expert and research teams to review current accounts of oncologists’ encounters with MD. Insights from these reviews contributed to the design of the semi-structured interview guide (Supplemental File 1).

The interview questions were designed to explore and evoke notions of how the personal beliefs, values, principles and practices of oncologists were influenced by their lived experiences in the field. Local oncology physicians and qualitative researchers then reviewed and refined the interview guide^44-46^ through a modified Delphi process.^47^

In 2023, 12 oncologists were recruited through email invitations sent to all medical oncologists in the National Cancer Centre Singapore, a tertiary cancer institute. Enclosed in the emails were the participant information sheets, including details on the study’s context, aims and duration. Participants were informed of their rights to anonymity and withdrawal from the study at any point without prejudice. Participation was also voluntary, with no incentives provided. Verbal and written consents were obtained from all participants before the commencement of the interviews. On an institutionally secured Zoom video-conferencing platform, two trained research team members conducted one-on-one interviews between 12th September 2023 and 17th December 2023. Each interview, conducted in English and audio-recorded with consent, lasted around an hour each. Data collection and analysis were conducted simultaneously. Three reviewers experienced in the use of Braun and Clarke’s^48^ approach to thematic analysis carried out independent analyses of the anonymised transcripts.

Participant recruitment and interviews were concluded when no new themes or insights emerged in two consecutive interviews. Audio recordings were anonymised and underwent transcription before member-checking for confirmation that the transcripts accurately conveyed the perceptions of the participants.

### Validity and Reliability of Analysis

For the purposes of triangulation, the secondary analysis was carried out by three independent reviewers (YTO, YY, VR). The codes and themes identified by each reviewer were then discussed with one another in online and face-to-face meetings. In addition, the findings of each reviewer and the subsequent combined analysis were reviewed by an experienced external reviewer well-versed in the topic at hand. To further ensure theoretical validation, the results of the analysis were compared with prevailing data. An iterative process was employed wherein the identification of any new codes prompted a review of all transcripts to verify classifications and ensure complete data extraction.

## Results

We analysed the results of our interviews with 12 practising Singaporean oncologists aged between 30-53 years, with a range of 4-13 years of specialist oncology experience. The demographics of the oncologists may be found in Table 1.

**Table 1.** Demographics of Interviewees.

Oncologist	Years of experience in oncology
A	10
B	7
C	8
D	Not mentioned
E	8
F	Not mentioned
G	Not mentioned
H	13
I	Not mentioned
J	4
K	4
L	Not mentioned

The following domains of key themes were found: 1) sources of MD; 2) sources of the costs of caring; 3) protective factors.

### Domain 1: Sources of Moral Distress

The oncologists revealed multiple sources of MD. Table 2 illustrates the various conflicts that hindered oncologists from practicing in accordance with their moral and ethical beliefs.

**Table 2.** Sources of Moral Distress.

Sources of conflict		Illustrative quote
Disagreements with Medical Team’s Decisions		If you work with doctors who don’t work with death and dying patients often, then there may be a little bit of disagreements. (Dr A)
		There were quite a few patients on TPN that were basically terminal… I didn’t feel that it was right… One or two of them had TPN all the way until they died. I did feel quite conflicted about that. (Dr H)
Disagreements with Patients’ Decisions		I do have people that I struggle with getting them to accept it’s time to go and to stop further treatment. (Dr E)
Disagreements with Patients’ Families		The common challenging issue I face is an ethical one about breaking the news to the patient because the family stands in the way… Sometimes the family will complain. (Dr G)
		There are certain instances where we are asked to assist or to support a patient’s life when we do not think that it is particularly meaningful or helpful. I still struggle with it intermittently. (Dr I)
Disagreements between Patient and Family		[Referring to 16-year-old patient] Mum felt that the treatment that we were offering was a lot more harmful to him and refused him to get chemotherapy… Eventual decision was still not to undergo the treatment… Sad thing was that the patient himself actually wanted the treatment. (Dr C)
Meeting Professional Expectations	Confronting Difficult Care Decisions	I really didn’t like to treat ovarian cancer patients because you can’t really do much. You just sit there and watch them slowly waste away and it’s not a nice death. (Dr E)
	Dealing with Emotive Situations	Young gentleman… within few weeks of this, his liver function just deteriorated so rapidly I couldn’t do anything about it, so I felt really, I still feel very helpless about it… It’s rough because he’s young, his family is very small and young too, things happening so quickly in short weeks. (Dr I)
		[I] cried in front of the family… felt so apologetic… worried that it was unprofessional. (Dr F)
	Difficulty Separating Between Professional and Personal Lives	In the past, I tended to bring thoughts about patients back home. I live alone so I have a lot of time to think about things I did wrong during the day. (Dr D)

### Domain 2: Sources of Costs of Caring

Iterative review of the data revealed several manifestations of emotional distress that extended beyond traditional notions of MD (Table 3).

**Table 3.** Sources of Costs of Caring.

Precipitants of the costs of caring	Illustrative quote
Palliative Sedation Leading to Compassion Fatigue	Did have one patient who was not ready to pass away and he was very traumatised… He had so much fear… Up to now, I still don’t know [whether sedating him was most humane way to let him live the rest of his life]. (Dr F)
Compromises to Care and Attention Leading to Compassion Fatigue and Burnout	I know some people who run like 30-40 patient full day clinics. When it’s so heavy, it’s really very difficult to have any meaningful conversation. (Dr A)
	I had a 19-year-old girl with lymphoma and was not responding to treatment. I was very close to her because whenever she got admitted, she always wanted to talk to me. There’s a bit of guilt because on her last day, she wanted to see me more often, but it wasn’t possible for me to be there so many times in a day. (Dr D)
Blurred Patient-Physician Boundaries Leading to Secondary Traumatic Stress and Vicarious Trauma	I had another young man who I was very close to… to the point where he was referred to as my baby brother. In a way, I didn’t know if I was overcompensating with him because I lost the other [another young patient] because he was also young. (Dr D)
Repeated and Prolonged Exposure to Death and Dying Leading to Compassion Fatigue and Burnout	If they’re really dying, then I’m the one who goes out and tells them, not for further treatment, I am the one who has to break it to them. Having a lot of conversations on end-of-life care caused this period of lacking empathy/compassion. (Dr D)
	You’re just delivering care like a robot. There’s just too many things, it becomes overwhelming and you’re just functioning at spinal cord level and just dishing out treatment without really engaging the patient. (Dr G)
	You feel constrained about your own mental load and you want to do other things, but you know that this person actually needs some help. But you feel like you can’t give any more than you’ve already given. (Dr B)
	[Referring to a patient who did not get chemotherapy due to familial conflicts] … Very first time that I actually thought I wanted to quit. (Dr C)

### Domain 3: Protective Factors

Oncologists reported a variety of methods to cope with MD and the costs of caring at both individual and institutional levels, as outlined in Table 4.

**Table 4.** Protective Factors.

Protective factors		Illustrative quote
Individual Level	Acceptance	Coming to terms and knowing it… it will drive you crazy [if you do not address MD]. (Dr K)
		By the time they see us, they may have a bit more symptoms and all. I feel it’s beyond my control, so I don’t really get too hung up over the inability to provide timely care… You just have to live with it, try again, change something. I realised I shouldn’t bank hopes on outcomes and rather focus on the process of doing it. (Dr G)
	Time-off	Need to get away… putting things down, putting work aside for a while, recalibrating and coming back to work. (Dr K)
	Compartmentalising	If I wasn’t coping well, I would’ve dropped out a long time ago… Oncology weeds out people that can’t manage the course… I’ve learnt not to be so affected by it. I still try to be sympathetic, but I don’t take it home with me. (Dr E)
	Self-awareness and Reflective Skills, Appreciating the Limits of their Influence	I have learned that I have to leave them [patients] to make their choice. If I give it one good go to convince them and they don’t follow my advice, I have learned I shouldn’t push it or feel guilty. The consequence of their decision shouldn’t be something I carry with me. (Dr D)
	Melding Personal and Professional Experiences	I have seen my mother go through cancer. So, post that experience, … I’m more accepting about what patients want for themselves. In the past, I tended to be more aggressive in pushing out treatment options… But sometimes, patients… don’t see that additional 6 months of survival as meaningful to them if they have to suffer side effects. And that’s extremely reasonable. (Dr H)
	Supportive Social Relationships	I thought I needed to go back to work but (boss) said take one more week. If I need time for myself, I am going to take it and it’s not selfish. It’s not unusual and it’s better overall for me to take that time. (Dr D)
Institutional Level	Role Modelling and Mentorship	Nowadays you have a department mentor…. An informal setting is the way to go. (Dr D)
	Training	Further training in the early stages of the career is certainly helpful…There was a [department] initiative—they recorded our breaking bad news conversation to a real patient and gave personalised feedback. I like that because it was direct and not so generic (Dr A)
	Establishing Supportive Work Culture	It’s just the people, the good people you work with… a supportive work culture is better than having structured programmes… It’s genuine. (Dr K)
	Increasing Manpower	The more manpower you have and the fewer cases you need to see in clinic, the more time we can spend…. to discuss and go through all their problems, not just restricted to the physical aspect of cancer. Now, the emotional, psychological, spiritual things are not addressed in clinic because you have 10 minutes. (Dr G)

## Discussion

This secondary analysis of semi-structured interviews with Singaporean oncologists carried out in light of widespread accounts of MD and the costs of caring has yielded several noteworthy insights.

**Figure 1.**
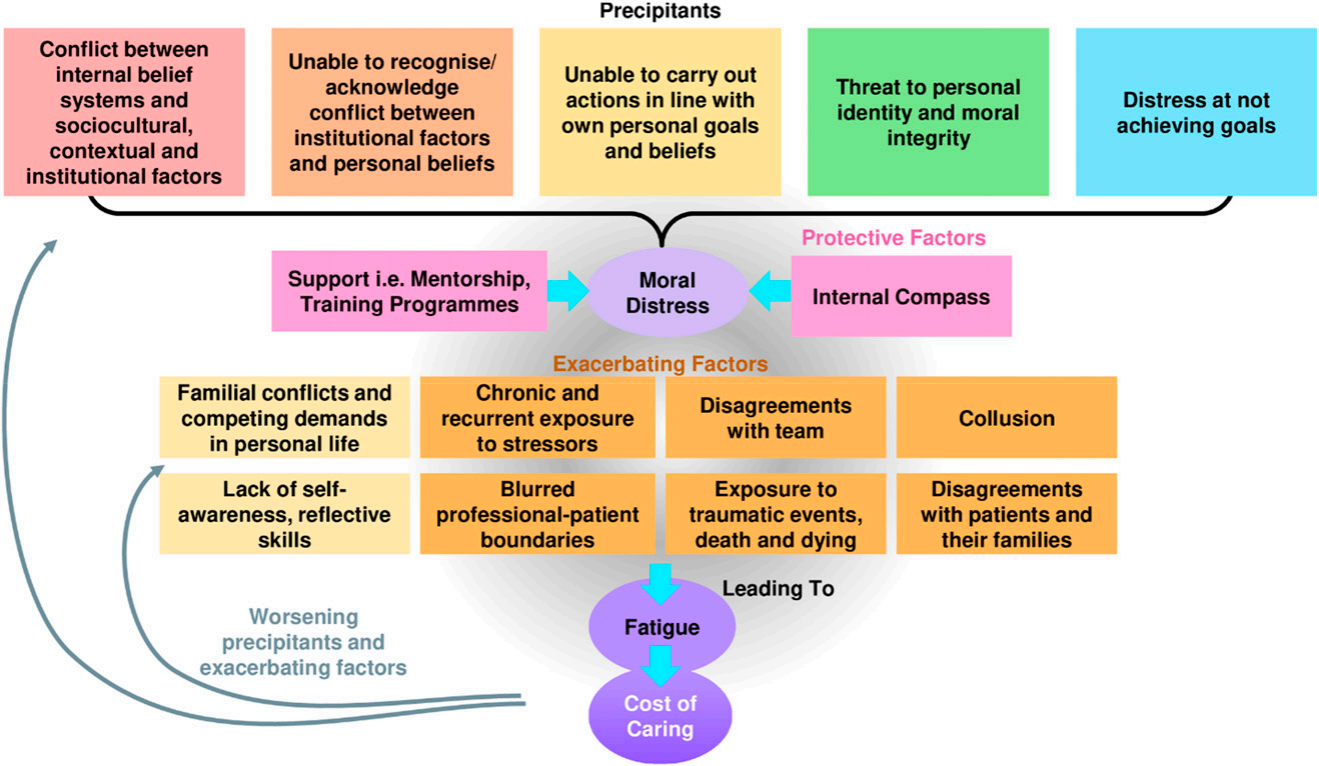
Factors influencing moral distress, fatigue and the cost of caring.

To begin, MD as a *“negative emotional response that occurs when physicians know the morally correct action but are prevented from taking it because of internal or external constraints”*^49^ is a layered and evolving sociocultural construct with deep ramifications upon oncologists. MD appears to be a highly personalised notion that extends beyond sociocultural considerations.^50^ Rather, it shows evolving individualised, relational, societal and psycho-emotional features linked to self-concepts of self and identity over time. If left unattended, it may lead to a wider cost of caring that encompasses CF, STS, VT culminating in burnout (Figure 1).^43,51^

First, it is proposed that MD may be triggered by multiple factors either on their own or, more likely, in combination with one another. This includes internal conflicts between professional roles and personal beliefs; conflicts between regnant belief systems and contextual considerations; inability to act in a manner that is consistent with the oncologist’s belief systems and ethical and moral perspectives; threats to the oncologist’s identity; and failure to achieve desired treatment objectives.

Acknowledgement that one or more sources may provoke MD raises the possibility of persistent or repeated episodes of MD, given that these triggers persist within the working environment.

Predisposing to MD are individual and contextual considerations. Individual considerations include compromises to familial and social support; lack of self-awareness and reflectivity; blurring of professional and personal boundaries through development of personal ties with patients and their families; and identification with patients and their circumstances. This may be further exacerbated by contextual considerations, such as the lack of effective support at work; high workloads; manpower and resource shortages; and a poor practice environment. Constant exposure to death and dying in an emotionally laden setting where there may be little mentoring, guided immersion, regular debriefs and reflective support amplifies the contextual considerations at play. These contextual factors also raise the possibility that they may co-exist with and even exacerbate individual considerations.

Conversely, attenuating the risk of MD and its sequalae are growing levels of resilience, developing experience, maturing competencies, deeper and more consistent reflectiveness, greater self-awareness and better coping and support mechanisms. These oncologist-specific factors are also complemented by better sensing of conflicts or situations that could raise MD. The ability to assess, adapt and re-evaluate situations further builds the oncologist’s *internal compass*, described as an overall moral code that guides decisioning and actions. A maturing *internal compass* and institutional support factors (denoted by the pink boxes in Figure 1) attenuate MD.

It is also through the secondary analysis that we find links between MD and physical and emotional exhaustion traditionally associated with compassion fatigue. The near constant exposure to traumatic events, associated with feelings of powerlessness in witnessing complicated clinical, psychosocial and existential needs of patients and their families, and contending with familial demands amidst high workloads and resource and manpower shortages perpetuate distress and give rise to secondary traumatic stress. Additionally, a blurring of the physician’s personal and professional boundaries derived from journeying and building personal ties with patients and their families predisposes to emotional distress or vicarious trauma. We refer to this unique blend of compassion fatigue, vicarious trauma, secondary traumatic stress and burnout as the costs of caring (denoted by dark purple oval in Figure 1). Such links between MD and these costs of caring are novel and reveal a feedback loop that sees the costs of caring reducing resilience, hampering the *internal compass* and accentuating the exacerbating factors featured in the yellow boxes in Figure 1.

The complexities brought to the fore by evaluating individual and contextual considerations underline the need for longitudinal support and mentored immersion into oncology where coping and effective decision-making can be taught and cultivated over the course of training and beyond. It also reveals the need for careful consideration of the practice environment and its hidden curriculum and culture. Furthermore, this theory of MD and its relationship with the broader costs of caring may be applied to other specialties and practice settings. As shown by recent studies, physicians do not have separate professional and personal selves, thus a holistic whole-physician approach is necessary to effectively support physicians.^12-14^

### Limitations

Interviewees who responded to our email invitations may have encountered more instances of MD or have been more impacted by MD. Interviewees who were more willing to share might also have more robust coping mechanisms that might not be representative of the oncologist population in Singapore. This generalisability of this study is further limited due to the exclusion of oncology trainees and other junior doctors caring for dying patients. Recall bias might pose a concern due to the one-off nature of the interviews.

## Conclusion

This study represents a call to action for host organisations. Evidence of MD and indeed its propensity to incur the costs of caring emphasises the need for better appreciation of this phenomenon. To facilitate efforts to enhance support of oncologists, further studies on MD through the lens of personhood are proposed as the effects of caring for terminally-ill patients extend beyond the professional sphere. The design of a personhood-based assessment tool of MD could complement longitudinal appraisals of the effects of MD to evaluate the complex interplay of individual and environmental considerations that precipitate the costs of caring. This then will be our focus of our coming research as we look forward to continuing our discussion on this critical intersection of staff wellbeing, patient safety and organisational reputation.

## Supplemental Material

Supplemental Material - Moral Distress and the Cost of Caring Amongst Medical Oncologists in Singapore Supplemental Material for Moral Distress and the Cost of Caring Amongst Medical Oncologists in Singapore by Varsha Rajalingam, Yutian Yu, Yun Ting Ong, Annushkha Sinnathamby, Nila Ravindran, Nagavalli Somasundaram, Simon Yew Kuang Ong, and Lalit Kumar Radha Krishna in American Journal of Hospice and Palliative Medicine.

## Data Availability

The authors confirm that the data supporting the findings of this study are available within the article and its supplementary materials. To request data from this study, please contact Prof Lalit Kumar Radha Krishna (lalit.radha-krishna@liverpool.ac.uk).

## Appendix 1

### Abbreviations

MD: Moral Distress
TPN: Total Parenteral Nutrition
